# Supplementary material for: COVID-19 Public Health Measures and Patient and Public Involvement in Health and Social Care Research: An Umbrella Review
Source: Int J Environ Res Public Health. 2023 Mar 10;20(6):4887. doi: 10.3390/ijerph20064887 (PMC10049580; doi:10.3390/ijerph20064887)
Supplement: Supplementary file 1 [file ijerph-20-04887-s001.zip › ijerph-2244711-supplementary.pdf]

Table of Contents

Supplementary Table S1. Search terms and search strategies........ 2
EBSCO databases search strategy........ 3
Epistemonikos........ 4
Google Scholar........ 5
ScienceDirect........ 5
World Health Organization COVID-19 database........ 5
Supplementary Table S2: Excluded articles with reasons........ 7
Supplementary Table S3: Assessment of methodological quality/critical appraisal of included studies...... 14
Supplementary Table S4: Summaries of included studies........ 15

Supplementary Table S1. Search terms and search strategies

PICo Concepts	Search terms	Notes
**Concept (P): Patient and Public Involvement**	(Citizen* OR Client* OR Carer* OR Community OR Consumer* OR Family OR Lay OR Participant* OR Participat* OR Patient* OR People OR Public* OR Stakeholder* OR Survivor* OR User*) AND ("Advisory Board" OR "Advisory Group" OR Advocacy OR Collaborat* OR Consult* OR "Consumer panel" OR Engag* OR Involv* OR Panel* OR Participat* OR Partner* OR Research OR "User led")	This search string is adapted from Rogers, M., Bethel, A., & Boddy, K. (2017). The development and testing of a Medline search filter to identify patient and public involvement in health research. Health Information & Libraries Journal, 34(2), 125-133.
**Concept (I): Public Health Measures**	"public health measures" OR "public health and social measures" OR "personal protective" OR "social measures" OR "social distancing" OR quarantin* OR isolation OR "contact tracing" OR lockdown OR isolation OR handwashing OR "washing hands" OR "cleaning hands" OR "mask wearing" OR "wearing a mask" OR "physical distancing" OR ((behavioral OR environmental OR social OR systems OR "non-pharmaceutical") AND (interventions OR measures)) OR "personal protective equipment" OR ((cleaning OR disinfecting) AND (surface* OR object*)) OR "air ventilation" OR "room humidification" OR "restricting entry" OR "restrict* travel" OR "biological measures"	Search terms were selected from the World Health Organization. (2020). Tracking Public Health and Social Measures: Taxonomy and Glossary. https://www.who.int/emergencies/diseases/novel-coronavirus-2019/phsm
**Concept (Co): COVID-19**	((wuhan AND (coronavirus OR corona virus)) OR (coronavirus* and ("19" or "2019")) OR COVID* OR nCov OR "novel coronavirus*" OR "novel corona virus*" OR "SARS-COV-2" OR "Severe Acute Respiratory Syndrome Coronavirus 2" OR "Severe Acute Respiratory Syndrome Corona virus 2" OR "coronavirus disease 2019" OR "corona virus disease 2019" OR "new coronavirus*" OR "new corona virus*" OR "SARS Coronavirus 2" OR "SARS Corona virus 2")	Adapted from CADTH COVID-19 search strings. (2021). https://covid.cadth.ca/literature-searching-tools/cadth-covid-19-search-strings/#covid-19-medline
**Concept used as a filter: Systematic review**	"systematic review" OR "systematic literature review" OR "systematic scoping review" OR "systematic narrative review" OR "systematic qualitative review" OR "qualitative systematic review" OR "qualitative evidence synthesis" OR "systematic evidence review" OR "systematic quantitative review" OR "systematic meta-review" OR "systematic critical review" OR "systematic search and review" OR "systematized review" OR "systematic mixed studies review" OR "mixed methods systematic review" OR "systematic mapping review" OR "systematic cochrane review" OR "systematic search and review" OR "systematic integrative review" OR "scoping review" OR "umbrella review" OR "systematic map" OR "mapping review" OR "meta-analysis" OR "rapid review" OR "state-of-the-art-review"	Adapted from National Library of Medicine. (2019). Search strategy used to create the PubMed systematic reviews filter. https://www.nlm.nih.gov/bsd/pubmed_subsets/sysreviews_strategy.html

EBSCO databases search strategy

TOTAL: 720 records

Academic Search Ultimate (152), APA PsychInfo (31), CINAHL (117), Family & Society Studies Worldwide (24), Health Source: Nursing/Academic Edition (22), MEDLINE (374)

Date: 1/21/2022

TI ((Citizen* OR Client* OR Carer* OR Community OR Consumer* OR Family OR Lay OR Participant* OR Participat* OR Patient* OR People OR Public* OR Stakeholder* OR Survivor* OR User*) AND ("Advisory Board" OR "Advisory Group" OR Advocacy OR Collaborat* OR Consult* OR "Consumer panel" OR Engag* OR Involv* OR Panel* OR Participat* OR Partner* OR Research OR "User led")) OR AB ((Citizen* OR Client* OR Carer* OR Community OR Consumer* OR Family OR Lay OR Participant* OR Participat* OR Patient* OR People OR Public* OR Stakeholder* OR Survivor* OR User*) AND ("Advisory Board" OR "Advisory Group" OR Advocacy OR Collaborat* OR Consult* OR "Consumer panel" OR Engag* OR Involv* OR Panel* OR Participat* OR Partner* OR Research OR "User led")) OR SU ((Citizen* OR Client* OR Carer* OR Community OR Consumer* OR Family OR Lay OR Participant* OR Participat* OR Patient* OR People OR Public* OR Stakeholder* OR Survivor* OR User*) AND ("Advisory Board" OR "Advisory Group" OR Advocacy OR Collaborat* OR Consult* OR "Consumer panel" OR Engag* OR Involv* OR Panel* OR Participat* OR Partner* OR Research OR "User led"))

AND

TI ("public health measures" OR "public health and social measures" OR "personal protective" OR "social measures" OR "social distancing" OR quarantin* OR isolation OR "contact tracing" OR lockdown OR isolation OR handwashing OR "washing hands" OR "cleaning hands" OR "mask wearing" OR "wearing a mask" OR "physical distancing" OR ((behavioural OR environmental OR social OR systems OR "non-pharmaceutical") N3 (interventions OR measures)) OR "personal protective equipment" OR ((cleaning OR disinfecting) AND (surface* OR object*)) OR "air ventilation" OR "room humidification" OR "restricting entry" OR "restrict* travel" OR "biological measures") OR AB ("public health measures" OR "public health and social measures" OR "personal protective" OR "social measures" OR "social distancing" OR quarantin* OR isolation OR "contact tracing" OR lockdown OR isolation OR handwashing OR "washing hands" OR "cleaning hands" OR "mask wearing" OR "wearing a mask" OR "physical distancing" OR ((behavioural OR environmental OR social OR systems OR "non-pharmaceutical") N3 (interventions OR measures)) OR "personal protective equipment" OR ((cleaning OR disinfecting) AND (surface* OR object*)) OR "air ventilation" OR "room humidification" OR "restricting entry" OR "restrict* travel" OR "biological measures") OR SU ("public health measures" OR "public health and social measures" OR "personal protective" OR "social measures" OR "social distancing" OR quarantin* OR isolation OR "contact tracing" OR lockdown OR isolation OR handwashing OR "washing hands" OR "cleaning hands" OR "mask wearing" OR "wearing a mask" OR "physical distancing" OR ((behavioural OR behavioral OR environmental OR social OR systems OR "non-pharmaceutical") N3 (interventions OR measures)) OR "personal protective equipment" OR ((cleaning OR disinfecting) AND (surface* OR object*)) OR "air ventilation" OR "room humidification" OR "restricting entry" OR "restrict* travel" OR "biological measures")

AND

TI ((wuhan AND (coronavirus OR corona virus)) OR (coronavirus* and ("19" or "2019")) OR COVID* OR nCov OR "novel coronavirus*" OR "novel corona virus*" OR "SARS-COV-2" OR "Severe Acute Respiratory Syndrome Coronavirus 2" OR "Severe Acute Respiratory Syndrome Corona virus 2" OR "coronavirus disease 2019" OR "corona virus disease 2019" OR "new coronavirus*" OR "new corona virus*" OR "SARS Coronavirus 2" OR "SARS Corona virus 2") OR AB ((wuhan AND (coronavirus OR corona virus)) OR (coronavirus* and ("19" or "2019")) OR COVID* OR nCov OR "novel coronavirus*" OR "novel corona virus*" OR "SARS-COV-2" OR "Severe Acute Respiratory Syndrome Coronavirus 2" OR "Severe Acute Respiratory Syndrome Corona virus 2" OR "coronavirus disease 2019" OR "corona

virus disease 2019" OR "new coronavirus*" OR "new corona virus*" OR "SARS Coronavirus 2" OR "SARS Corona virus 2") OR SU ((wuhan AND (coronavirus OR corona virus)) OR (coronavirus* and ("19" or "2019")) OR COVID* OR nCov OR "novel coronavirus*" OR "novel corona virus*" OR "SARS-COV-2" OR "Severe Acute Respiratory Syndrome Coronavirus 2" OR "Severe Acute Respiratory Syndrome Corona virus 2" OR "coronavirus disease 2019" OR "corona virus disease 2019" OR "new coronavirus*" OR "new corona virus*" OR "SARS Coronavirus 2" OR "SARS Corona virus 2")

AND

TI ("systematic review" OR "systematic literature review" OR "systematic scoping review" OR "systematic narrative review" OR "systematic qualitative review" OR "qualitative systematic review" OR "qualitative evidence sythesis" OR "systematic evidence review" OR "systematic quantitative review" OR "systematic meta-review" OR "systematic critical review" OR "systematic search and review" OR "systematized review" OR "systematic mixed studies review" OR "mixed methods systematic review" OR "systematic mapping review" OR "systematic cochrane review" OR "systematic search and review" OR "systematic integrative review" OR "scoping review" OR "umbrella review" OR "systematic map" OR "mapping review" OR "meta-analysis" OR "rapid review" OR "state-of-the-art-review") OR AB ("systematic review" OR "systematic literature review" OR "systematic scoping review" OR "systematic narrative review" OR "systematic qualitative review" OR "qualitative systematic review" OR "qualitative evidence sythesis" OR "systematic evidence review" OR "systematic quantitative review" OR "systematic meta-review" OR "systematic critical review" OR "systematic search and review" OR "systematized review" OR "systematic mixed studies review" OR "mixed methods systematic review" OR "systematic mapping review" OR "systematic cochrane review" OR "systematic search and review" OR "systematic integrative review" OR "scoping review" OR "umbrella review" OR "systematic map" OR "mapping review" OR "meta-analysis" OR "rapid review" OR "state-of-the-art-review") OR SU ("systematic review" OR "systematic literature review" OR "systematic scoping review" OR "systematic narrative review" OR "systematic qualitative review" OR "qualitative systematic review" OR "qualitative evidence sythesis" OR "systematic evidence review" OR "systematic quantitative review" OR "systematic meta-review" OR "systematic critical review" OR "systematic search and review" OR "systematized review" OR "systematic mixed studies review" OR "mixed methods systematic review" OR "systematic mapping review" OR "systematic cochrane review" OR "systematic search and review" OR "systematic integrative review" OR "scoping review" OR "umbrella review" OR "systematic map" OR "mapping review" OR "meta-analysis" OR "rapid review" OR "state-of-the-art-review")

Epistemonikos

https://www.epistemonikos.org/

TOTAL: 357 records

Date: 1/21/2022

((Citizen* OR Client* OR Carer* OR Community OR Consumer* OR Family OR Lay OR Participant* OR Participat* OR Patient* OR People OR Public* OR Stakeholder* OR Survivor* OR User*) AND ("Advisory Board" OR "Advisory Group" OR Advocacy OR Collaborat* OR Consult* OR "Consumer panel" OR Engag* OR Involv* OR Panel* OR Participat* OR Partner* OR Research OR "User led")) **AND** ("public health measures" OR "public health and social measures" OR "personal protective" OR "social measures" OR "social distancing" OR quarantin* OR isolation OR "contact tracing" OR lockdown OR isolation OR handwashing OR "washing hands" OR "cleaning hands" OR "mask wearing" OR "wearing a mask" OR "physical distancing" OR ((behavioural OR behavioral OR environmental OR social OR systems OR "non-pharmaceutical") AND (interventions OR measures)) OR "personal protective equipment" OR ((cleaning OR disinfecting) AND (surface* OR object*)) OR "air ventilation" OR "room humidification" OR "restricting entry" OR "restrict* travel" OR "biological measures") **AND** (((wuhan AND (coronavirus OR corona virus)) OR

(coronavirus* and ("19" or "2019")) OR COVID* OR nCov OR "novel coronavirus*" OR "novel corona virus*" OR "SARS-COV-2" OR "Severe Acute Respiratory Syndrome Coronavirus 2" OR "Severe Acute Respiratory Syndrome Corona virus 2" OR "coronavirus disease 2019" OR "corona virus disease 2019" OR "new coronavirus*" OR "new corona virus*" OR "SARS Coronavirus 2" OR "SARS Corona virus 2")) [Filters: protocol=no, classification=systematic-review, min_year=2020, max_year=2022]

Note: Searched in *Title/Abstract* - advanced search box - then limited to "*Systematic review*"

Google Scholar

TOTAL: 45 records

Date: 1/21/2022

("patient and public involvement" OR "patient and public engagement") **AND** ("public health measures" OR "public health and social measures") **AND** (COVID OR SARS-CoV-2)

Note: Limited to review articles from the left side menu

ScienceDirect

TOTAL: 11 records

Date: 1/21/2022

(“patient and public involvement” OR “patient and public engagement”) **AND** (“public health measures” OR “public health and social measures”)

World Health Organization COVID-19 database

https://search.bvsalud.org/global-literature-on-novel-coronavirus-2019-ncov/

TOTAL: 288 records

Date: 1/21/2022

(tw:((Citizen* OR Client* OR Carer* OR Community OR Consumer* OR Family OR Lay OR Participant* OR Participat* OR Patient* OR People OR Public* OR Stakeholder* OR Survivor* OR User*) AND ("Advisory Board" OR "Advisory Group" OR Advocacy OR Collaborat* OR Consult* OR "Consumer panel" OR Engag* OR Involv* OR Panel* OR Participat* OR Partner* OR Research OR "User led"))) **AND** (tw:("public health measures" OR "public health and social measures" OR "personal protective" OR "social measures" OR "social distancing" OR quarantin* OR isolation OR "contact tracing" OR lockdown OR isolation OR handwashing OR "washing hands" OR "cleaning hands" OR "mask wearing" OR "wearing a mask" OR "physical distancing" OR ((behavioral OR environmental OR social OR systems OR

"non-pharmaceutical") AND (interventions OR measures)) OR "personal protective equipment" OR ((cleaning OR disinfecting) AND (surface* OR object*)) OR "air ventilation" OR "room humidification" OR "restricting entry" OR "restrict* travel" OR "biological measures")) **AND** (tw:(((wuhan AND (coronavirus OR corona virus)) OR (coronavirus* and ("19" or "2019")) OR COVID* OR nCov OR "novel coronavirus*" OR "novel corona virus*" OR "SARS-COV-2" OR "Severe Acute Respiratory Syndrome Coronavirus 2" OR "Severe Acute Respiratory Syndrome Corona virus 2" OR "coronavirus disease 2019" OR "corona virus disease 2019" OR "new coronavirus*" OR "new corona virus*" OR "SARS Coronavirus 2" OR "SARS Corona virus 2"))) **AND** (tw:("systematic review" OR "systematic literature review" OR "systematic scoping review" OR "systematic narrative review" OR "systematic qualitative review" OR "qualitative systematic review" OR "qualitative evidence synthesis" OR "systematic evidence review" OR "systematic quantitative review" OR "systematic meta-review" OR "systematic critical review" OR "systematic search and review" OR "systematized review" OR "systematic mixed studies review" OR "mixed methods systematic review" OR "systematic mapping review" OR "systematic cochrane review" OR "systematic search and review" OR "systematic integrative review" OR "scoping review" OR "umbrella review" OR "systematic map" OR "mapping review" OR "meta-analysis" OR "rapid review" OR "state-of-the-art-review"))

Supplementary Table S2: Excluded articles with reasons

No	Excluded Studies	Reason for Exclusion
1	Acharya, A., Judah, G., Ashrafian, H., Sounderajah, V., Johnstone-Waddell, N., Stevenson, A., & Darzi, A. (2021). Investigating the implementation of SMS and Mobile Messaging in population Screening (the SIPS Study): Protocol for a Delphi Study. *JMIR Research Protocols*, *10*(12), e32660. https://doi.org/10.2196/32660	Results have not been published yet. The authors confirmed this is not a systematic review.
2	Akinbi, A., Forshaw, M., & Blinkhorn, V. (2021). Contact tracing apps for the COVID-19 pandemic: A systematic literature review of challenges and future directions for neo-liberal societies. *Health Information Science and Systems*, *9*(1), 18. https://doi.org/10.1007/s13755-021-00147-7	Does not mention PPI
3	AlAmodi, A. A., Al-Kattan, K., & Shareef, M. A. (2021). The current global perspective of the knowledge-attitude-behavior of the general public towards the corona virus disease -19 pandemic: Systematic review and meta-analysis on 67,143 participants. *PLOS ONE*, *16*(12), e0260240. https://doi.org/10.1371/journal.pone.0260240	Does not mention PPI
4	Baines, R., Tredinnick-Rowe, J., Jones, R., & Chatterjee, A. (2020). Barriers and Enablers in implementing electronic consultations in primary care: Scoping review. *Journal of Medical Internet Research*, *22*(11), e19375. https://doi.org/10.2196/19375	Does not mention PPI
5	Betancourt, J. A., Rosenberg, M. A., Zevallos, A., Brown, J. R., & Mileski, M. (2020). The Impact of COVID-19 on telemedicine utilization across multiple service lines in the United States. *Healthcare*, *8*(4), 380. https://doi.org/10.3390/healthcare8040380	Does not mention PPI
6	Bevan, I., Link to external site, this link will open in a new window, Baxter, M. S., Stagg, H. R., Link to external site, this link will open in a new window, Street, A., & Link to external site, this link will open in a new window. (2021). Knowledge, attitudes, and behavior related to COVID-19 testing: A rapid scoping review. *Diagnostics*, *11*(9), 1685. https://doi.org/10.3390/diagnostics11091685	Does not mention PPI
7	Biddle, M. S. Y., Gibson, A., & Evans, D. (2021). Attitudes and approaches to patient and public involvement across Europe: A systematic review. *Health & Social Care in the Community*, *29*(1), 18–27. https://doi.org/10.1111/hsc.13111	Not COVID-19 related
8	Brooks, S. K., Greenberg, N., Wessely, S., & Rubin, G. J. (2021). Factors affecting healthcare workers' compliance with social and behavioural infection control measures during emerging infectious disease outbreaks: Rapid evidence review. *BMJ Open*, *11*(8), e049857. https://doi.org/10.1136/bmjopen-2021-049857	Does not mention PPI

No	Excluded Studies	Reason for Exclusion
9	Carter, P., Megnin-Viggars, O., & Rubin, G. J. (2021). What factors influence symptom reporting and access to healthcare during an emerging infectious disease outbreak? A rapid review of the evidence. *Health Security*, *19*(4), 353–363. https://doi.org/10.1089/hs.2020.0126	Does not mention PPI
10	Chen, J., & Wang, Y. (2021). Social media use for health purposes: Systematic review. *Journal of Medical Internet Research*, *23*(5), e17917. https://doi.org/10.2196/17917	Does not mention PPI
11	Clavel, N., Badr, J., Gautier, L., Lavoie-Tremblay, M., & Paquette, J. (2021). Risk perceptions, knowledge and behaviors of general and high-risk adult populations towards COVID-19: A systematic scoping review. *Public Health Reviews*, *42*, 1603979. https://doi.org/10.3389/phrs.2021.1603979	Does not mention PPI
12	De Freitas, L., Basdeo, D., & Wang, H.-I. (2021). Public trust, information sources and vaccine willingness related to the COVID-19 pandemic in Trinidad and Tobago: An online cross-sectional survey. *The Lancet Regional Health - Americas*, *3*, 100051. https://doi.org/10.1016/j.lana.2021.100051	Not a review article
13	Fong, V. C., & Iarocci, G. (2020). Child and family outcomes following pandemics: A systematic review and recommendations on COVID-19 policies. *Journal of Pediatric Psychology*, *45*(10), 1124–1143. https://doi.org/10.1093/jpepsy/jsaa092	Does not mention PPI
14	Ghio, D., Lawes-Wickwar, S., Tang, M. Y., Epton, T., Howlett, N., Jenkinson, E., Stanescu, S., Westbrook, J., Kassianos, A. P., Watson, D., Sutherland, L., Stanulewicz, N., Guest, E., Scanlan, D., Carr, N., Chater, A., Hotham, S., Thorneloe, R., Armitage, C. J., … Keyworth, C. (2021). What influences people's responses to public health messages for managing risks and preventing infectious diseases? A rapid systematic review of the evidence and recommendations. *BMJ Open*, *11*(11), e048750. https://doi.org/10.1136/bmjopen-2021-048750	Does not mention PPI
15	Gilmore, B., Ndejjo, R., Tchetchia, A., De Claro, V., Mago, E., Lopes, C., & Bhattacharyya, S. (2020). Community engagement for COVID-19 prevention and control: a rapid evidence synthesis. *BMJ Global Health*, *5*(10), e003188. http://dx.doi.org/10.1136/bmjgh-2020-003188	Not COVID-19 related
16	Iqbal, M. P., Walpola, R., Harris-Roxas, B., Li, J., Mears, S., Hall, J., & Harrison, R. (2021). Improving primary health care quality for refugees and asylum seekers: A systematic review of interventional approaches. Health Expectations. https://doi.org/10.1111/hex.13365	Not COVID-19 related

No	Excluded Studies	Reason for Exclusion
17	Jamal, Z., Perkins, A., Allen, C., Evans, R., Sturgess, J., Snowdon, C., Clayton, T., Elbourne, D., & Research Advisory Group. (2021). Patient and public involvement prior to trial initiation: Lessons learnt for rapid partnership in the COVID-19 era. *Research Involvement and Engagement*, *7*(1), 13. https://doi.org/10.1186/s40900-021-00250-9	Not a review article
18	Kasar, K. S., & Karaman, E. (2021). Life in lockdown: Social isolation, loneliness and quality of life in the elderly during the COVID-19 pandemic: A scoping review. *Geriatric Nursing*, *42*(5), 1222–1229. https://doi.org/10.1016/j.gerinurse.2021.03.010	Does not mention PPI
19	Khorram-Manesh, A., Dulebenets, M. A., & Goniewicz, K. (2021). Implementing public health strategies—the need for educational initiatives: A systematic review. *International Journal of Environmental Research and Public Health*, *18*(11), 5888. https://doi.org/10.3390/ijerph18115888	Does not mention PPI
20	Lai, S. H. S., Tang, C. Q. Y., Kurup, A., & Thevendran, G. (2021). The experience of contact tracing in Singapore in the control of COVID-19: Highlighting the use of digital technology. *International Orthopaedics*, *45*(1), 65–69. https://doi.org/10.1007/s00264-020-04646-2	Does not mention PPI
21	Liyanage, P., Rocklöv, J., & Tissera, H. A. (2021). The impact of COVID–19 lockdown on dengue transmission in Sri Lanka; A natural experiment for understanding the influence of human mobility. *PLOS Neglected Tropical Diseases*, *15*(6), e0009420. https://doi.org/10.1371/journal.pntd.0009420	Does not mention PPI
22	Maqbool, A., & Khan, N. Z. (2020). Analyzing barriers for implementation of public health and social measures to prevent the transmission of COVID-19 disease using DEMATEL method. *Diabetes & Metabolic Syndrome: Clinical Research & Reviews*, *14*(5), 887–892. https://doi.org/10.1016/j.dsx.2020.06.024	Does not mention PPI
32	Massey, D., Huang, C., Lu, Y., Cohen, A., Oren, Y., Moed, T., Matzner, P., Mahajan, S., Caraballo, C., Kumar, N., Xue, Y., Ding, Q., Dreyer, R., Roy, B., & Krumholz, H. (2021). Engagement with COVID-19 public health measures in the United States: A cross-sectional social media analysis from June to November 2020. *Journal of Medical Internet Research*, *23*(6), e26655. https://doi.org/10.2196/26655	Does not mention PPI
24	Monteiro, K. S., Santino, T. A., Jácome, A. C., Silva, B., Patino, C. M., Chaves, G., Alchieri, J. C., Leite, S., Luz, K. G., Guerra, R. O., & Mendonça, K. M. P. P. de. (2021). Barriers and facilitators to populational adherence to prevention and control measures of COVID-19 and other respiratory infectious diseases: A rapid qualitative evidence synthesis protocol. *BMJ Open*, *11*(1), e045529. https://doi.org/10.1136/bmjopen-2020-045529	Does not mention PPI

No	Excluded Studies	Reason for Exclusion
25	Moran, C., Campbell, D. J. T., Campbell, T. S., Roach, P., Bourassa, L., Collins, Z., Stasiewicz, M., & McLane, P. (2021). Predictors of attitudes and adherence to COVID-19 public health guidelines in Western countries: A rapid review of the emerging literature. *Journal of Public Health*, *43*(4), 739–753. https://doi.org/10.1093/pubmed/fdab070	Does not mention PPI
26	Moss, S. J., Krewulak, K. D., Stelfox, H. T., Ahmed, S. B., Anglin, M. C., Bagshaw, S. M., Burns, K. E. A., Cook, D. J., Doig, C. J., Fox-Robichaud, A., Fowler, R., Hernández, L., Kho, M. E., Kredentser, M., Makuk, K., Murthy, S., Niven, D. J., Olafson, K., Parhar, K. K. S., … Fiest, K. M. (2021). Restricted visitation policies in acute care settings during the COVID-19 pandemic: A scoping review. *Critical Care*, *25*(1), 347. https://doi.org/10.1186/s13054-021-03763-7	Does not mention PPI
27	Nagel, A., Łaszewska, A., Haidinger, G., & Simon, J. (2021). The first 8 weeks of the Austrian SARS-CoV-2 epidemic. *Wiener Klinische Wochenschrift*, *133*(7), 364–376. https://doi.org/10.1007/s00508-020-01804-9	Not a review article
28	Nochaiwong, S., Ruengorn, C., Awiphan, R., Ruanta, Y., Boonchieng, W., Nanta, S., Kowatcharakul, W., Pumpaisalchai, W., Kanjanarat, P., Mongkhon, P., Thavorn, K., Hutton, B., Wongpakaran, N., & Wongpakaran, T. (2020). Mental health circumstances among health care workers and general public under the pandemic situation of COVID-19 (HOME-COVID-19). *Medicine*, *99*(26), e20751. https://doi.org/10.1097/MD.0000000000020751	Not a review article
29	Nochaiwong, S., Ruengorn, C., Thavorn, K., Hutton, B., Awiphan, R., Phosuya, C., Ruanta, Y., Wongpakaran, N., & Wongpakaran, T. (2021). Global prevalence of mental health issues among the general population during the coronavirus disease-2019 pandemic: A systematic review and meta-analysis. *Scientific Reports*, *11*(1), 10173. https://doi.org/10.1038/s41598-021-89700-8	Does not mention PPI
30	Nordin, N. R. M., Arsad, F. S., Kamaruddin, P. S. N. M., Hilmi, M., Madrim, M. F., Hassan, M. R., Rahim, S. S. S. A., Jeffree, M. S., Ramdzan, A. R., & Atil, A. (2021). Impact of social distancing on COVID-19 and other related infectious disease transmission: A systematic review. *Open Access Macedonian Journal of Medical Sciences*, *9*(F), 601–607.	Does not mention PPI
31	Nussbaumer-Streit, B., Mayr, V., Dobrescu, A. I., Chapman, A., Persad, E., Klerings, I., Wagner, G., Siebert, U., Ledinger, D., Zachariah, C., & Gartlehner, G. (2020). Quarantine alone or in combination with other public health measures to control COVID-19: A rapid review. *Cochrane Database of Systematic Reviews*, *9*. https://doi.org/10.1002/14651858.CD013574.pub2	Does not mention PPI

No	Excluded Studies	Reason for Exclusion
32	O'Donovan, M., Buckley, C., Benson, J., Roche, S., McGowan, M., Parkinson, L., Byrne, P., Rooney, G., Bergin, C., Walsh, D., Bird, R., McGroarty, F., Fogarty, H., Smyth, E., Ahmed, S., O'Donnell, J. S., Ryan, K., O'Mahony, B., Dougall, A., & O'Connell, N. M. (2020). Telehealth for delivery of haemophilia comprehensive care during the COVID-19 pandemic. *Haemophilia*, *26*(6), 984–990. https://doi.org/10.1111/hae.14156	Not a review article
33	O'Leary, N., Kingston, L., Griffin, A., Morrissey, A., Noonan, M., Kelly, D., Doody, O., Niranjan, V., Gallagher, A., O'riordan, C., & Lynch, A. (2021). COVID-19 healthcare policies in Ireland: A rapid review of the initial pandemic response. *Scandinavian Journal of Public Health*, *49*(7), 713–720. https://doi.org/10.1177/14034948211008371	Does not mention PPI
34	O'Sullivan, L., Killeen, R. P., Doran, P., & Crowley, R. K. (2021). Adherence with reporting of ethical standards in COVID-19 human studies: A rapid review. *BMC Medical Ethics*, *22*(1), 80. https://doi.org/10.1186/s12910-021-00649-9	Does not mention PPI
35	Pai, N., & Vella, S.-L. (2021). COVID-19 and loneliness: A rapid systematic review. *Australian & New Zealand Journal of Psychiatry*, *55*(12), 1144–1156. https://doi.org/10.1177/00048674211031489	Does not mention PPI
36	Piovani, D., Christodoulou, M. N., Hadjidemetriou, A., Pantavou, K., Zaza, P., Bagos, P. G., Bonovas, S., & Nikolopoulos, G. K. (2021). Effect of early application of social distancing interventions on COVID-19 mortality over the first pandemic wave: An analysis of longitudinal data from 37 countries. *Journal of Infection*, *82*(1), 133–142. https://doi.org/10.1016/j.jinf.2020.11.033	Not a review article
37	Pires, C. (2021). A pre-systematic review on the use of masks as a protection material for SARS-COV-2 during the COVID-19 pandemic. *International Journal of Clinical Practice*, *75*(9), e14215. https://doi.org/10.1111/ijcp.14215	Does not mention PPI
38	Polisena, J., Ospina, M., Sanni, O., Matenchuk, B., Livergant, R., Amjad, S., Zoric, I., Haddad, N., Morrison, A., Wilson, K., Bogoch, I., & Welch, V. A. (2021). Public health measures to reduce the risk of SARS-CoV-2 transmission in Canada during the early days of the COVID-19 pandemic: A scoping review. *BMJ Open*, *11*(3), e046177. https://doi.org/10.1136/bmjopen-2020-046177	Does not mention PPI
39	Radfar, A., Caceres, M. M. F., Sosa, J. P., & Filip, I. (2021). Overcoming the challenges of the mental health care system in United States in the aftermath of COVID-19. *CNS Spectrums*, *26*(2), 176–176. https://doi.org/10.1017/S1092852920002886	Does not mention PPI

No	Excluded Studies	Reason for Exclusion
40	Raynaud, M., Zhang, H., Louis, K., Goutaudier, V., Wang, J., Dubourg, Q., Wei, Y., Demir, Z., Debiais, C., Aubert, O., Bouatou, Y., Lefaucheur, C., Jabre, P., Liu, L., Wang, C., Jouven, X., Reese, P., Empana, J.-P., & Loupy, A. (2021). COVID-19-related medical research: A meta-research and critical appraisal. *BMC Medical Research Methodology*, *21*(1), 1. https://doi.org/10.1186/s12874-020-01190-w	Does not mention PPI
41	Regmi, K., & Lwin, C. M. (2020). *Factors impacting social distancing measures for preventing coronavirus disease 2019 [COVID-19]: A systematic review.* https://doi.org/10.21203/rs.3.rs-37498/v1	Does not mention PPI
42	Regmi, K., & Lwin, C. M. (2021). Factors associated with the implementation of non-pharmaceutical interventions for reducing coronavirus disease 2019 (COVID-19): A systematic review. *International Journal of Environmental Research and Public Health*, *18*(8), 4274. https://doi.org/10.3390/ijerph18084274	Does not mention PPI
43	Ryan, R. E., Parkhill, A., Schonfeld, L., Walsh, L., Lowe, D., Merner, B., Nelson, N., & Hill, S. J. (2021). *What are relevant, feasible and effective approaches to promote acceptance, uptake and adherence to physical distancing measures for COVID-19 prevention and control?* World Health Organization. Regional Office for Europe. https://apps.who.int/iris/handle/10665/339887	Does not mention PPI
44	Salari, N., Khazaie, H., Hosseinian-Far, A., Khaledi-Paveh, B., Kazeminia, M., Mohammadi, M., Shohaimi, S., Daneshkhah, A., & Eskandari, S. (2020). The prevalence of stress, anxiety and depression within front-line healthcare workers caring for COVID-19 patients: A systematic review and meta-regression. *Human Resources for Health*, *18*(1), 100. https://doi.org/10.1186/s12960-020-00544-1	No public health measures
45	Seligman, W. H., Fialho, L., Sillett, N., Nielsen, C., Baloch, F. M., Collis, P., Demedts, I. K. M., Fleck, M. P., Floriani, M. A., Gabriel, L. E. K., Gagnier, J. J., Keetharuth, A., Londral, A., Ludwig, I. I. L., Lumbreras, C., Daza, A. M., Muhammad, N., Bastos, G. A. N., Owen, C. W., … Brinkman, K. (2021). Which outcomes are most important to measure in patients with COVID-19 and how and when should these be measured? Development of an international standard set of outcomes measures for clinical use in patients with COVID-19: a report of the International Consortium for Health Outcomes Measurement (ICHOM) COVID-19 Working Group. *BMJ Open*, *11*(11), e051065. https://doi.org/10.1136/bmjopen-2021-051065	No public health measures
46	Sepúlveda-Loyola, W., Rodríguez-Sánchez, I., Pérez-Rodríguez, P., Ganz, F., Torralba, R., Oliveira, D. V., & Rodríguez-Mañas, L. (2020). Impact of social isolation due to COVID-19 on health in older people: Mental and physical effects and recommendations. *The Journal of Nutrition, Health & Aging*, *24*(9), 938–947. https://doi.org/10.1007/s12603-020-1500-7	Does not mention PPI

No	Excluded Studies	Reason for Exclusion
47	Shi, M., Stelick, A., Licker, S., & Dando, R. (2021). On the validity of longitudinal comparisons of central location consumer testing results prior to COVID-19 versus home use testing data during the pandemic. *Journal of Food Science*, *86*(10), 4668–4677. https://doi.org/10.1111/1750-3841.15905	Does not mention PPI
48	Viner, R., Russell, S., Saulle, R., Croker, H., Stansfeld, C., Packer, J., Nicholls, D., Goddings, A.-L., Bonell, C., Hudson, L., Hope, S., Schwalbe, N., Morgan, A., & Minozzi, S. (2021). *Impacts of school closures on physical and mental health of children and young people: A systematic review* (p. 2021.02.10.21251526). medRxiv. https://doi.org/10.1101/2021.02.10.21251526	Does not mention PPI

Supplementary Table S3: Assessment of methodological quality/critical appraisal of included studies

Authors (year)	Q1	Q2	Q3	Q4	Q5	Q6	Q7	Q8	Q9	Q10	Q11	Risk of Bias
Adebisi et al. (2021) [23]	Yes	Yes	Yes	Yes	Yes	Yes	Unclear	Unclear	No	Yes	Unclear	Medium
Banerjee et al. (2021) [24]	Yes	Yes	Unclear	No	N/A	N/A	Unclear	Yes	Unclear	Yes	Yes	Medium
Ernawati et al. (2021) [25]	Yes	Yes	Yes	Yes	Yes	Unclear	No	N/A	No	No	No	Medium
Mao et al. (2021) [26]	Yes	Yes	Yes	Yes	Yes	Yes	No	Unclear	Yes	Yes	Yes	Low
Pegollo et al. (2021) [27]	Yes	Yes	Yes	Yes	Yes	Yes	Yes	Yes	Yes	Yes	Yes	Low
Raymond & Ward (2021) [28]	Yes	Yes	Yes	Yes	Yes	Yes	No	Yes	Yes	Yes	Yes	Low

Questions:

Q1. Is the review question clearly and explicitly stated?
Q2. Were the inclusion criteria appropriate for the review question?
Q3. Was the search strategy appropriate?
Q4. Were the sources and resources used to search for the studies adequate?
Q5. Were the criteria for appraising studies appropriate?
Q6. Was critical appraisal conducted by two or more reviewers independently?
Q7. Were there methods to minimize errors in data extraction?
Q8. Were the methods used to combine the studies appropriate?
Q9. Was the likelihood of publication bias assessed?
Q10. Were recommendations for policy and/or practice supported by the reported data?
Q11. Were the specific directives for new research appropriate?

Risk of bias scores

- Low risk of bias (0-3)
- Medium risk of bias (4-6)
- High risk of bias (7-11)

Note: The "Risk of Bias" column is a result of the total number of points scored for each appraisal question.

Supplementary Table S4: Summaries of included studies

Authors	Adebisi et al. (2021) [23]	Banerjee et al. (2021) [24]	Ernawati et al. (2021) [25]	Mao et al. (2021) [26]	Pegollo et al. (2021) [27]	Raymond & Ward (2021) [28]
**Objective(s)**	Catalog the risk communication and community engagement (RCCE) strategies as well as the challenges facing RCCE in 13 African countries.	Systematically review patient and public involvement and engagement (PPIE) in the development of mortality risk tools and availability and use of patient-facing mortality risk information, and interactively develop, with patients, accessible mortality risk information for "high-risk" conditions for COVID-19.	Determine the community's knowledge, attitudes, and behavior in preventing the transmission of COVID-19.	Learn about COVID-19 volunteering both at the UK national level and the more local community level.	Investigate population acceptance for digital contract tracing (DCT). DCT needs population acceptance, which is influenced by digital literacy, attitudes, and practice, in order to be effective.	Identify what is currently known about how communities in low- and middle-income countries (LMICs) are responding to the pandemic, the socio-cultural context of each selected LMIC, and theories pertaining to each LMICs public health design.
**Definition of Patient and Public Involvement (PPI)**	RCCE refers to "the processes and approaches to systematically consult, engage, and communicate with communities who are at risk, or whose practices affect risk". The aim of RCCE is to urge, enable, and include stakeholders in the prevention of and response to public health emergencies.	N/A	Community participation is a sincere willingness of the community to help with disease management activities in their respective regions so that the spread of COVID-19 does not expand. Therefore, people with their awareness do social distancing and self-isolation.	N/A	N/A	N/A

Authors	Adebisi et al. (2021) [23]	Banerjee et al. (2021) [24]	Ernawati et al. (2021) [25]	Mao et al. (2021) [26]	Pegollo et al. (2021) [27]	Raymond & Ward (2021) [28]
**Definition of Public Health Measures (PHM)**	Contact tracing and points of entry screening, disease prevention and control in healthcare facilities, laboratory diagnosis, and the clinical management of COVID-19 patients.	N/A	N/A	N/A	Population acceptance is a key component of DCT effective adoption and, ultimately, infection control during infectious diseases outbreaks.	LMICs that are currently faced with unprecedented social ruptures, restrictions in social and physical mobility, and ever-looming uncertainties of infection, financial insecurity, stigma, and loss, communities worldwide reacted in multiple and complex ways. Additionally, LMICs that are heavily subjected to misinformation and fear of social rejection resulted in noncompliance with pandemic sanctions, resistance, and increased isolation, allowing the spread of the disease. Likewise, the meaning of and understandings about COVID-19 were constructed using traditional, religious, and biomedical epistemologies, which were occasionally in conflict with each other.

Authors	Adebisi et al. (2021) [23]	Banerjee et al. (2021) [24]	Ernawati et al. (2021) [25]	Mao et al. (2021) [26]	Pegollo et al. (2021) [27]	Raymond & Ward (2021) [28]
**Participants (characteristics/ total number)**	Unclear	29.1 million patients from 76 countries	Total of research samples: 18,897	Some list N/A, others include 36, 539, 7, 57, 31890, 317, 20, 94, 854, 62, 4827, 13, 182<br>Community groups, volunteering groups, volunteers, or community champions	- 186,144 surveyed subjects<br>- 50,000 tweets<br>- 5,025 Reddit posts<br>- 714 written comments	N/A
**Setting/ context**	In 2020, the WHO provided guidance on RCCE for countries to help protect the people's health in response to the outbreak. A total of 10 actionable plans were recommended on how to develop effective RCCE strategies in preparation for the outbreak. However, unproductive RCCE in some African countries still threatened an effective response to the pandemic.	PPIE faces challenges due to COVID-19. Risk tools have emerged but may not directly respond to patient and public concerns or provide publicly available baseline mortality risk data across a wide range of conditions.	Questionnaire	Various countries mainly in England, Scotland, and the United Kingdom	General population, as well as specific populations and underrepresented minorities	Government mandated restrictions on movement to reduce transmission rates in lower/middle income nations
**Interventions/ phenomena of interest**	Risk communication and community engagement (RCCE) strategies	COVID-19 risk prediction tools	Knowledge, attitudes, and behaviors, of communities in prevention of COVID-19 transmission	COVID-19 volunteering in the UK	Digital contact tracing (DCT)	Government mandated restrictions in relation to social and public health adaptive measures

Authors	Adebisi et al. (2021) [23]	Banerjee et al. (2021) [24]	Ernawati et al. (2021) [25]	Mao et al. (2021) [26]	Pegollo et al. (2021) [27]	Raymond & Ward (2021) [28]
**Search details/ keywords/ search terms**	"community engagement," "outbreak communication," "risk communication," "social mobilization," "health education," "health promotion," "crisis communication," "COVID-19," "2019-nCoV," "SARS-CoV-2," "{+Each one of the 13 African Countries}	N/A	Search terms: knowledge, attitude, practice, and COVID-19 transmission	("COVID-19" OR "Coronavirus") AND ("volunteering" OR "mutual aid" OR "community" OR "community engagement" OR "community champions")	Free text and MeSH terms in the following concepts:<br>1. Digital contact tracing (DCT)<br>2. COVID-19 pandemic	"COVID*" AND "ethnograph*" OR "anthropology" OR "qualitative", and variations that included terms such as "case study", "phenomenology", "lived experience", "meaning", "study", "research", and "empirical". These terms were used to ensure broad capture of the available literature.
**Number of databases/ sources searched**	MEDLINE, PubMed Central, PubMed, Google Scholar, and Google.	PubMed and Google Scholar	PLOS ONE, Pro-Quest, and Google Scholar.	ScienceDirect, University of Sussex Library, APA Psycnet, Wiley Online Library, PubMed and SocArXiv	PubMed, Embase, Web of Science and the Cochrane Library	The databases used include Anthrosource, Google Scholar, Ovid, Pubmed, Proquest Social Science, Scopus, and Web of Science.
**Date range of included studies in the systematic reviews**	December 2019 and February 2021	January 2020 - February 2021	January-July 2020	January-October 2020	2020 onwards	December 2019-August 2021
**Number of studies included**	N/A	53 published COVID-19 risk prediction tools	10	27	41	25

Authors	Adebisi et al. (2021) [23]	Banerjee et al. (2021) [24]	Ernawati et al. (2021) [25]	Mao et al. (2021) [26]	Pegollo et al. (2021) [27]	Raymond & Ward (2021) [28]
**Types of studies included**	Peer reviewed articles, reports, newsletters, government documents	Systematic review of COVID-19 risk prediction tools, generation, and presentation of observed mortality risks in underlying conditions, focus on patient and public involvement and engagement, informing the prototype	Quantitative research, primary data, open access articles, peer-reviewed	Published peer-reviewed articles, reports, briefings, blog posts, newspaper articles, and online media	Cross-sectional, population-based controlled experiment, surveys, interviews, text analysis, readability, experiments, longitudinal, comparative mixed methods, app review analysis, app usability, hybrid, and prospective	Empirical, qualitative, field-based, and/or participatory research
**Country of origin of included studies**	Algeria, Ghana, South Africa, Tanzania, Kenya, Mauritius, Angola, Cote d'Ivoire, Ethiopia, the Democratic Republic of the Congo, Nigeria, Zambia, and Uganda	United Kingdom	India, China, unknown for rest	United Kingdom	**1. Europe** - Belgium, France, Germany, Iceland, Ireland, Italy, Netherlands, Poland, Spain, Switzerland, United Kingdom<br>**2. Australia and New Zealand**<br>**3. America** - Argentina, Brazil, Canada, Colombia, United States<br>**4. Asia** - Bahrain, India, Israel, Japan, Singapore, Taiwan<br>**5. Africa** - Ghana, South Africa	Bolivia, Burkina Faso, Ethiopia, Ghana, Indonesia, Nepal, Nigeria, Pakistan, Philippines, Sierra Leone, and Uganda

Authors	Adebisi et al. (2021) [23]	Banerjee et al. (2021) [24]	Ernawati et al. (2021) [25]	Mao et al. (2021) [26]	Pegollo et al. (2021) [27]	Raymond & Ward (2021) [28]
**Appraisal instrument(s) and rating**	Unclear	N/A	Unclear	Mixed methods appraisal tool (MMAT)<br>Studies were rated as:<br>- Good quality if they scored four or more out of five;<br>- Moderate quality if they scored three out of five;<br>- Poor quality if they scored two or less out of five.	RTI item bank	JBI critical appraisal tools for qualitative research<br>Studies were excluded due to low methodological rigor, geographic ineligibility, or an ineligible study focus area.
**Type of review**	Narrative review	Systematic review and mixed method	Systematic review	Rapid review of the literature	Systematic review	Systematic review
**Outcome(s) assessed**	RCCE strategies to address COVID-19 pandemic in 13 African countries	Involvement of patients and public in existing mortality risk information for people with diseases considered high risk for COVID-19	Number of research samples used/the number of item categories in the aspects assessed by each factor; disease modes of transmission, common systems, and prevention	What have we learned from COVID-19 volunteering both at the UK national level and the local community level? Answering this question may also illuminate political, organizational, and psychological aspects of COVID-19 volunteering that will be useful for responses to future disease outbreaks	Digital contact tracing (DCT) acceptance determinants in different settings, populations, and cultural contexts	Government mandated restrictions for movement in relation to social and psychological effects such as fear, anxiety, shame, and stigma amongst community members, patients, and healthcare workers

Authors	Adebisi et al. (2021) [23]	Banerjee et al. (2021) [24]	Ernawati et al. (2021) [25]	Mao et al. (2021) [26]	Pegollo et al. (2021) [27]	Raymond & Ward (2021) [28]
**Results/ Findings**	Most of the WHO-prioritized African countries have RCCE strategies to curb the spread of COVID-19. However, the RCCE response activities were not without challenges, which included distrust in the government, cultural, social, and religious resistance, and inertia, as well as widespread fake news and rumors, the exclusion of vulnerable populations and longstanding issues of weak healthcare systems.	This study shows a lack of public and patient engagement in COVID-19 risk tools to date and a lack of mortality risk information design for patients with underlying conditions. Throughout the pandemic, a sustained patient and public interest and engagement in developing a risk information tool during and beyond the pandemic was demonstrated.	Many respondents had good knowledge of COVID-19	The findings suggest that food shopping and emotional support were the most common activities, but there were diverse models of organization and coordination in COVID-19 volunteering. Additionally, community support groups seem to be adjusting their activities and scope of action to current needs and challenges. Furthermore, despite the efforts of a few official public institutions and councils, there has been limited community engagement and collaboration with volunteering groups and other community-based organizations.	Early adopters display pro-socialness traits, higher COVID-19 risk perception, and more technological fluency. Late adopters are incidentally those that would need the most protection but often lack the equipment and understanding of technology Interventions are speculative but transparent communication, education-level-adjusted, aimed at explaining the technology and the boundaries on data usage is fundamental.	Analyzed reactive responses, social and psychological effects, the impacts of social and mobility restrictions, health system challenges, and adaptive responses. Found increased feelings of rejection, exhaustion, and isolation from healthcare workers, patients, caregivers, and patients who were at high-risk of infection. Poverty and inequality served as barriers to complying with the lockdown orders
**Significance/ direction**	Based on results, researchers recommend strengthening the strategic mapping of partners, investing in proper coordination structures, resources, and training, improving public trust through	The Montgomery ruling places a duty on doctors to provide 'all material risks' when faced with consenting patients, but new ways to generate and communicate reliable risk information are required, with wide applications	Community knowledge determines how people will behave in relation to the pandemic	Further research should focus on how organizations, including both official and non-official bodies, can improve their collaboration and cooperation with volunteer-based groups, in order to foster community	Assessing DCT acceptance's determinants in different settings, populations, and cultural contexts is of fundamental importance to inform the planning, implementation, and monitoring of public health interventions.	Community engagement is imperative in implementing and following public health measures. These measures were influenced by political instability, economics, religion, policy, and structural influences

Authors	Adebisi et al. (2021) [23]	Banerjee et al. (2021) [24]	Ernawati et al. (2021) [25]	Mao et al. (2021) [26]	Pegollo et al. (2021) [27]	Raymond & Ward (2021) [28]
	effective leaderships, ensuring adequate planning, and strengthening documentation and the reporting of activities and experiences for RCCE in Africa.	beyond COVID-19. There is a role for charities, patient organizations, and patients to come together in order to articulate a framework of understanding the demands for better risk information across disease silos.		engagement and participation		
